# Parent–daughter emotional dyssynchrony correlates with personality and psychopathology in adolescents with anorexia nervosa

**DOI:** 10.1007/s40519-025-01726-3

**Published:** 2025-02-28

**Authors:** Federico Amianto, Jessica Maria Angelini, Chiara Davico, Daniele Marcotulli, Antonella Anichini, Elena Rainò, Benedetto Vitiello

**Affiliations:** 1 Departmento of Neuroscience, Section of Child and Adolescent Neuropsychiatry, University of Turin, Turin, Italy; 2 Department of Public Health and Pediatric Sciences, Section of Child and Adolescent Neuropsychiatry, University of Turin, Turin, Italy; 3 Department of Biomedical, Metabolic and Neural Sciences, Section of Child and Adolescent Neuropsychiatry, University of Modena and Reggio-Emilia, Modena, Italy

**Keywords:** Adolescence, Anorexia nervosa, Relational attunement, Dyssynchrony, Personality, Psychopathology

## Abstract

**Purpose:**

Anorexia nervosa (AN) is a major concern in adolescents. Attachment problems contribute to its pathogenesis and maintenance. This research explores the relationship between parent–daughter emotional dyssynchrony and the psychopathology of AN daughters.

**Methods:**

One hundred and fourteen female adolescents with AN and their parents participated in this study. The daughters completed the youth self-report (YSR) and other self-report measures of personality, eating, and general psychopathology. The parents completed the Child Behavior Checklist (CBCL). The YSR and CBCL ratings were compared and the difference was considered a measure of emotional dyssynchrony. Dyssynchrony scores were correlated with daughters’ personality and psychopathology.

**Results:**

Mothers scored lower on 15/21 (71%), and fathers scored lower on 11/21 (52%). Mothers scored higher than fathers did in thought problems. Mothers' dyssynchrony was positively related to their daughters' harm avoidance and negatively related to their self-directedness. Both parents' dyssynchrony correlated with their daughters' eating habits and general psychopathology. Dyssynchrony in each psychopathological measure of YSR was positively correlated with specific daughters’ psychopathological traits.

**Conclusions:**

Parents' perceptions underestimate the suffering of adolescents with AN. Mothers’ perception was worse than that of fathers, possibly because of greater overinvolvement. Parent–daughter dyssynchrony is largely related to daughters’ psychopathology. This cross-sectional study could not confirm whether dyssynchrony contributed to the onset of AN or follows it. Interventions that promote emotional synchronization may aid in the treatment of adolescents with AN.

**Level of evidence:**

III.

## Introduction

Anorexia nervosa (AN) is a complex disorder resulting from multiple risk factors related to social, individual, psychological, familial, and biological aspects [1].

Among the factors involved in AN pathogenesis, the role of the family remains controversial. Along with the psychodynamic interpretation of the disease [2], some authors consider a dysfunctional mother–daughter relationship as a primary cause of AN [3], whereas others attribute it to only a marginal role [4].

Emotional attunement, neurobiologically represented by the mirror neurons [5], is an intersubjective space shared by mother and infant since birth that leads the child to building its identity [6]. Through the mirror neurons activation, the observer's behavior is attuned to the emotional expression of the subject with whom he/she interacts. Some authors [7] describe attunement as a process by which the mother's brain affects the child’s brain by linking it to social participation. If emotional attunement is impaired, the infant's empathic behavior will be reduced, as will his/her emotional coping with life stressors in adolescence, fourfolds increasing the risk of psychopathology [8].

Through these considerations, the defective structure of the self in AN daughters [9] may be related to a reduced capacity of emotional regulation and attunement [10], within a family environment perceived as unsuitable for learning to understand and manage emotional states [3].

The current literature that analyses the emotional relationship between parents and daughters mainly focuses on the role of attachment, identifying how AN correlates with an insecure style [11, 12], where individuals are highly likely to be unable to relate adequately, and implement coping strategies to oppose a sense of distress, such as accentuating their feelings (through guilt) or suppressing them [13].

Among the correlation between the development of AN and deficit parental emotional attunement [3], specific research is scarce.

The aim of the present study was to expand the knowledge about the correlation between mother–daughter and father–daughter emotional dyssynchrony and the symptom expression of AN adolescents. Dyssynchrony was first used in a study of children with Prader–Willy syndrome [14], which demonstrated the positive effects of oxitocine administration in improving mother–child attachment. The definition of emotional dyssynchrony is reciprocal to emotional attunement. The authors preferred to use this term because with the applied methodology is more realistic that they evidenced the negative, e.g., partial, with respect to the positive face of the emotional attunement, which is a wider and more complex construct. The guiding hypothesis is that greater dyssynchrony in adolescence relates to worse psychopathological symptoms expressed by the AN adolescent daughter. The authors considered as a measure of dyssynchrony the difference in the same subscales filled-in by parents in the Child Behavior Checklist (CBCL), and by daughters in the Youth Self Report (YSR), paired instruments assessing children psychopathology and general functioning [14]. Dyssynchrony quantifies parents’ ability to perceive their daughters’ emotional states and suffering. Then it was correlated with self-rated measures of personality, attachment and psychopathology, commonly used in the clinical assessment of AN because related to its pathogenesis and the clinical evolution [15]. The hypothesis is that parent–daughter dyssynchrony may directly correlate with personality, attachment and psychopathology features of AN adolescents. Nevertheless, dyssynchrony may be caused by both parents’ and childrens’ problems [14]. Moreover, the present study is a cross-sectional study; therefore, no causal hypothesis was considered in the interpretation of the results.

## Methods

### Participants

The recruited sample included 130 female adolescents with typical AN or atypical AN who were referred to the Outpatient Services for Adolescent Psychopathology and Eating Disorders of Regina Margherita Children's Hospital in Turin. Participants were assessed by a neuropsychiatrist using the K-SADS for DSM-5 Disorders [16] along with a non-structured interview with parents.

Inclusion criteria: female sex; age between 12 and 18 years; completeness of psychometric tests by the daughter and at least one parent; AN or atypical AN diagnosis; absence of mental retardation or pervasive neurodevelopmental disorders; parents’ consent. Sixteen female adolescents were excluded.

The final group included 114 adolescents (BMI range: 12.9–22.9): full AN diagnosis = 92 (85 restrictive, 7 binge-purging) and atypical AN = 22 (21 restrictive, 1 binge-purging). The samples did not differ significantly in clinical and psychopathological measures, and they were considered as a whole. None of the participants was treated with drugs.

One-hundred-ten mothers and 35 fathers (age range: 35–59 years) were included in the analyses.

### Ethics

Written informed consent was obtained from both parents and daughters. This study was conducted in accordance with the principles of the Declaration of Helsinki, as revised in October 2013. The inter-company review board of Torino (CEI) approved this study (protocol 00007/2019; protocol number 0099307).

### Assessment procedure and measures

During the first visit, the following self-administered inventories were sent to both the adolescents and parents.

#### Youth self-report (YSR) and child behavior checklist (CBCL)

The YSR is a questionnaire filled out by patients, and the CBCL is the version filled out by the parents [17].

They measure adolescents’ social skills and emotional–behavioral problems in the previous 6 months. The empirically based scales are: anxious/depressed, withdrawn/depressed, somatic complaints, social problems, thought problems, attention problems, rule-breaking behavior, aggressive behavior, internalizing problems, externalizing problems, total problems. Cronbach’s alpha for the Italian version ranging from 0.78 (somatic complaints and thought problems) to 97 (total problems) for both the tests.

The DSM-oriented scales include affective, anxiety, somatic, ADHD, oppositional defiant, and conduct problems. Cronbach’s alpha for the Italian version ranging from 0.78 (anxiety problems) to 91 (conduct problems) for both the tests. The Italian version is available at: http://www.aseba.org.

#### Temperament and character inventory (TCI)

The TCI consists of 240 dichotomous-response items [18]. It analyzes seven dimensions of personality, four of these investigate temperament which describe the innate characteristics of personality, including novelty seeking (NS), harm avoidance (HA), reward dependence (RD), and persistence (P). Character, which describes personality characteristics acquired through interactions with the environment during development, includes self-directedness (SD), cooperativeness (C), and self-transcendence (ST). The Cronbach’s alpha for the Italian version was 0.72.

#### Eating disorder inventory 2 (EDI-2)

The EDI-2 [19] measures disordered eating attitudes, behaviors, and personality traits common to individuals diagnosed with eating disorders. It assessed the main eating psychopathological features related to eating disorders (drive to thinness, bulimia, body dissatisfaction, inadequacy, perfectionism, interpersonal distrust, interoceptive awareness, fear of maturity, asceticism, impulsiveness, and social insecurity). The Cronbach’s alpha for the Italian version was 0.81.

#### Symptom check-list-90 (SCL-90)

The SCL-90 [20] investigates the most common psychological or psychosomatic symptoms that the patient may have suffered in the past or at the time of compilation of the test. A global operating index, called the global score index, and nine primary symptomatic dimensions were calculated: somatization, obsessive–compulsive, interpersonal sensitivity, depression, anxiety, hostility, phobic anxiety, paranoid ideation, and psychoticism. The Cronbach’s alpha for the Italian version was 0.96.

This test was included to explore general AN adolescents’ psychopathology dimensions independently from the YSR administration and to correlate them with the YSR–CBCL differences.

#### Toronto alexithymia scale (TAS-20)

The TAS-20 was used to evaluate the presence of alexithymia, considering three main features [21]: feelings identification, feeling description, and outward-oriented thinking. The Cronbach’s alpha for the Italian version was 0.89.

#### Attachment style questionnaire (ASQ)

The ASQ [22] evaluates the current attachment style discriminating between secure and insecure attachments. The ASQ is a 40-item questionnaire that measure five scales: confidence (in self and others), discomfort with closeness, need for approval, preoccupation with relationships and relationships as secondary.

### Statistical analysis

Only the CBCL of parents was included in the analysis, whereas all tests filled-in by daughters were included.

Daughters’ YSR and parents’ CBCL for each subscale were compared among the mother–daughter, father–daughter, and mother–father couples using the Student’s *t* test to determine their dyssynchrony.

The absolute differences between each corresponding subscale (named “delta”, Δ) were considered measures of dyssynchrony. The sum of the Δ of all subscales (total Δ) was computed and considered the overall measure of parent–daughter dyssynchrony. The total Δ was correlated with the clinical and psychometric measures of the daughters using Pearson’s correlation analysis. The Δ correlation with the YSR/CBCL was not considered, because Δ computation was based on these tests. Instead, each Δ was correlated with the daughter’s YSR psychopathology using a multivariate correlation analysis to account for the interactions between multiple dependent variables.

Due to the large number of variables examined, Bonferroni correction was applied to the *t* test analysis with a *p* < 0.002 significance to reduce the risk of type I errors. Owing to the explorative nature of the study and the data reduction obtained in the first analysis, a *p* < 0.01 was considered acceptable for statistical significance in the multivariate analysis of variance (MANOVA) analysis.

Statistical analyses were performed using Statistical Package for the Social Sciences (SPSS 27.0, 2021).

## Results

### CBCL–YSR mother–daughter comparison

Table 1 compares the daughter's YSR test results with those of the mother's CBCL test, the daughters’ YSR test results with the father's CBCL test results and mothers’ CBCLs with fathers’ CBCLs.

**Table 1:** Mother–daughter, father–daughter and mother–father comparison

Daughters YSR–mothers CBCL	PT *N* = 114 mn ± sd	*M* *N* = 110 mn ± sd	*t*	*p*
Accademic performances	2.31 ± 0.48	4.36 ± 1.61	− 14.242	0.000
Anxious/depressed	11.77 ± 5.82	8.50 ± 4.52	5.337	0.000
Social complaints	4.63 ± 3.59	3.43 ± 3.09	3.947	0.000
Social problems	3.94 ± 3.12	2.17 ± 2.26	6.704	0.000
Thoughts problems	3.95 ± 4.01	2.68 ± 2.97	3.955	0.000
Attention problems	6.27 ± 3.28	4.02 ± 3.16	7.264	0.000
Aggressive behavior	8.37 ± 4.16	5.97 ± 3.89	5.534	0.000
Internalizing problems	22.15 ± 10.92	16.89 ± 8.25	4.917	0.000
Externalizing problems	10.79 ± 6.20	8.04 ± 5.47	4.491	0.000
Total problems	52.49 ± 24.24	38.28 ± 18.07	6.523	0.000
Anxiety problems	6.07 ± 3.06	3.95 ± 2.46	3.857	0.000
Attention and hyperactivity problems	5.12 ± 2.81	2.98 ± 2.76	5.872	0.000
Oppositional defiant problems	4.51 ± 1.85	2.68 ± 1.88	5.556	0.000
Obsessive–compulsive problems	7.71 ± 4.34	4.63 ± 2.88	4.649	0.000
Post-traumatic stress problems	13.54 ± 5.63	9.41 ± 40.00	5.315	0.000

Daughters YSR–fathers CBCL	PT *N* = 114 mn ± sd	*F* *N* = 35 mn ± sd	*t*	*p*
Anxious/depressed	120.00 ± 6.82	7.53 ± 5.56	3.501	0.001
Social problems	5.38 ± 3.03	2.81 ± 2.15	3.695	0.001
Thoughts problems	6.75 ± 5.04	3.09 ± 2.99	3.871	0.001
Attention problems	6.56 ± 3.40	3.69 ± 3.86	4.104	0.000
Aggressive behavior	8.50 ± 3.91	5.10 ± 4.02	4.324	0.000
Internalizing problems	25.74 ± 13.93	13.87 ± 10.70	4.658	0.000
Externalizing problems	11.47 ± 5.63	6.82 ± 5.67	4.347	0.000
Total problems	60.35 ± 27.48	34.42 ± 23.24	5.171	0.000
Oppositional defiant problems	4.22 ± 1.70	2.41 ± 1.66	5.574	0.000
Obsessive–compulsive problems	7.41 ± 4.80	3.81 ± 3.21	3.786	0.001
Post-traumatic stress problems	12.72 ± 6.04	7.91 ± 5.85	3.966	0.000

Mother–father CBCL	*M* *N* = 110 mn ± sd	*F* *N* = 35 mn ± sd	*t*	*p*
Thought problems	5.61 ± 3.25	3.13 ± 3.03	3.790	0.001

YSR: Youth Self-report; CBCL: Child Behavior Checklist; PT: patient sample; N: sample size; mn: mean; sd: standard deviation; F: father sample; M: mother sample

Mothers scored higher than daughters in academic performance and lower in anxiety/depressed problems, social complaints, social problems, thought problems, attention problems, aggressive behavior, internalizing problems, externalizing problems, total problems, anxiety problems, attention and hyperactivity problems, oppositional defiant problems, obsessive–compulsive problems, and post-traumatic stress problems (15/21, 71% of the scales, all *p* < 0.001).

Fathers scored lower than daughters did on anxious/depressed problems, social problems, thought problems, attention problems, aggressive behavior, internalizing problems, externalizing problems, total problems, oppositional defiant problems, obsessive–compulsive problems, and post-traumatic stress problems (11/21, 52% of the scales, all *p* < 0.001).

Mothers scored higher on the thought problems scale than fathers (*p* < 0.001).

### Pearson’s correlation between mother’s dyssynchrony and daughter’s psychopathology, father’s dyssynchrony and daughter’s psychopathology

Total maternal dyssynchrony was positively correlated with daughters’ harm avoidance (*p* < 0.001), drive to thinness (*p* < 0.006), bulimia (*p* < 0.001), body dissatisfaction (*p* < 0.001), inadequacy (*p* < 0.001), perfectionism (*p* < 0.005), interoceptive awareness (*p* < 0.001), asceticism (*p* < 0.001), impulsiveness (*p* < 0.001), and social insecurity (*p* < 0.001), and negatively related to self-directedness (*p* < 0.004) (Table 2).

**Table 2:** Pearson’s correlation

Total mother–daughter Δ	*r*	*p*
*Temperament and Character Inventory*		
Harm avoidance	0.386	0.000
Self-directedness	− 0.288	0.004
*Eating Disorder Inventory-2*		
Drive to thinness	0.263	0.006
Bulimia	0.327	0.001
Body dissatisfaction	0.327	0.001
Perfectionism	0.268	0.005
Inadequacy	0.441	0.000
Interoceptive awareness	0.372	0.000
Ascetism	0.335	0.000
Impulsiveness	0.380	0.000
Social insecurity	0.378	0.000

Total father–daughter Δ		
Weekly vomiting frequency	0.508	0.005
*Eating Disorder Inventory-2*		
Inadequacy	0.514	0.002

Δ = difference between daughters’ YSR and parents’ CBCL o parents’ CBCL; df = 34

Total paternal dyssynchrony was positively correlated with the daughters’ weekly vomiting frequency (*p* < 0.005) and inadequacy (*p* < 0.002) (Table 2).

### MANOVA correlation of parent dyssynchrony with daughters’ psychopathology subscales

Mother–daughter dyssynchrony in academic performance was correlated with body dissatisfaction (*p* < 0.005), interoceptive awareness (*p* < 0.002), and externally oriented thinking (*p* < 0.001).

Mother–daughter dyssynchrony in social complaints correlated with preoccupation with relationships (*p* < 0.001), ability to describe feelings (*p* < 0.009), and total alexithymia (*p* < 0.006).

Mother–daughter dyssynchrony in attention problems correlated with externally oriented thinking (*p* < 0.004) and total alexithymia (*p* < 0.002).

Mother–daughter dyssynchrony in aggressive behavior correlated with somatization (*p* < 0.009).

Mother–daughter dyssynchrony in externalizing problems was correlated with somatization (*p* < 0.004) and preoccupation with relationships (*p* < 0.002). Mother–daughter dyssynchrony in total problems correlated with daughter’s self-directedness (*p* < 0.004) (Table 3).

**Table 3:** MANOVA correlation

Mother–daughter Δ	Correlated variables	*F*	*p*
Δ Accademic performances	Body dissatisfaction^a^	8.326	0.005
	Interoceptive awareness^a^	10.295	0.002
	Externally oriented thinking^b^	15.633	< 0.001
Δ Social complaints	Preoccupation with relationship^c^	12.653	0.001
	Describing feelings^b^	7.973	0.009
	Total alexithymia^b^	8.967	0.006
Δ Attention problems	Externally oriented thinking^b^	9.777	0.004
	Total alexithymia^b^	11.263	0.002
Δ Aggressive behavior	Somatization^d^	7.834	0.009
Δ Externalizing problems	Somatization^d^	9.756	0.004
	Preoccupation with relationship^c^	12.035	0.002
Total problems	Self-directedness^e^	8.640	0.004

Δ = difference between daughters’ YSR and parents’ CBCL

^a^Eating Disorder Inventory-2

^b^Toronto Alexithymia Scale-20

^c^Attachment Style Questionnaire

^d^Symptom Cecklist-90

^e^Temperament and Character Inventory

## Discussion

Dyssynchrony with parents may receive different interpretations, not mutually exclusive. First, parental dyssynchrony may have exerted a pathogenic role: parents’ difficulty to take a perspective-taking empathy towards their daughters may have influenced attachment dynamics [2], hampering the development of the self, and facilitating the development of AN [11]. Second, the onset of the AN may have hindered parents in emphasizing with their daughters’ suffering due to the incomprehensibility of ED attitudes. Third, even daughters’ internalizing attitudes and alexithymic traits may have impaired parents’ reading of their emotional states [23]. Fourth, it may represent an attempt to conceal adolescents’ emotional states from caregivers during the individuation process. The cross-sectional design of the study impairs any inference regarding the direction or causal effect of the correlation.

Mothers had a very high rate of completion of the CBCL, whereas fathers completed the questionnaire in only 31% of cases, suggesting that mothers’ involvement in their daughters’ suffering is higher than the father’s. Nevertheless, studies comparing daughter–parent assessments using the same instruments are scarce. No study included only female adolescents; those who included both female and male samples reported a similar lack of convergence, particularly for internalizing scales [24, 25]. In a study conducted on male and female adolescents affected by substance disorder (SUDs) [26], parent–son convergence ranged from 70 to 90%, with the highest convergence in the scale of conduct problems, mothers displayed a slightly lower completion rate than in the present study (90%), but it was not possible to compare fathers, because only parents alone were included. In contrast, another one [27] conducted on male adolescents with SUD, demonstrated significant differences between son and parents in externalizing problems, aggressive behaviors, and social attention problems, but no difference for most internalizing problems. This suggests that in the same clinical population, there is high variability in parent–son dyssynchrony, possibly due to sampling biases and/or cultural differences. As a common report, all studies on parent–son dyssynchrony provide evidence that parents tend to overestimate the psychopathology traits of their sons [24–27]. Instead, a new finding of this study is that only academic performance was overestimated by mothers, while all the other psychopathology and functioning scales were underestimated.

Usually, in AN, mothers experience high levels of subjective caregiver burden [28], and their perception of their daughter is related to their degree of over-involvement [29]. Nevertheless, no study has previously measured the ability of AN adolescents’ mothers to perceive their daughters’ suffering. The exploration of parent–daughter convergence in the assessment of family functioning evidenced that mothers tend to underestimate family malfunctioning with respect to their daughters and fathers [30]; it is possible that mother–daughter dyssynchrony may be a self-defense of mothers’ over-involvement with AN daughters’ suffering [28, 29].

Personality traits of parents of AN daughters are influential on parental coping strategies for management of AN and thus may be helpful to interpret the present results [31]. In the previous studies, mothers and fathers’ immature personality traits were supposed to contribute to the development of daughters’ AN [32]. The only personality trait which distinguished both parents with respect to the normative population was the low self-transcendence. This personality function permits the perception of the inner states of others with perspective-taking empathy [28, 33, 34]. This kind of empathy, may be raised by family counselling/therapy interventions [30].

Differentiation between maternal and paternal liability on the development of personality and psychopathology of AN daughters has already been evidenced in a previous study [32]. It supports a specific relational dynamic linking parents’ dyssynchrony to daughters’ psychopathological expressions.

This is the first evidence of a correlation between daughter–mother dyssynchrony with two personality traits (harm avoidance and self-directedness) considered the “personality core” of AN [35] and risk factors for AN development in siblings developing within the same family context [36]. According to the previously bi-directional hypothesis, it is possible that maternal dyssynchrony may have fostered high harm avoidance [37] and low self-directedness [38]. However, on the contrary, these traits may foster an avoidant mother–daughter relationship impairing emotional attunement. Neuroimaging studies indicate that impaired emotional attachment with the caregiver during development negatively affects the maturation of brain structures [39]. The critical review by Scalabrini and coworkers [40], relates some neuroimaging evidence concerning the lack of emotional attunement, impaired early attachment, and inadequate caregiving to the development of the relational brain. This, in turn, is related to the level of personality organization and refers to the integration of the self: a lack of self-integration is one of the most relevant risk factors for the development of AN [11]. The board relationship between maternal dyssynchrony and daughters’ eating psychopathology could be related to these evidences, also because it is specific to paternal dyssynchrony. Moreover, a previous study on parental invalidation [41] supports the possible role of maternal invalidation of the daughters’ emotional experiences in the onset of eating symptoms. On the other hand, the development of eating psychopathology may have reduced the mother’s ability to synchronize with the daughter.

The correlation between paternal dyssynchrony and frequency of vomiting in daughters is novel and suggestive and supports a previous research [42] which correlated the rate of vomiting to paternal invalidation in the ED, suggesting that parental attitudes may directly influence this eating symptom.

When the dyssynchrony scores in each dimension of the YSR/CBCL tests correlated with the daughters’ measures, a more complex and detailed picture emerged: also alexithymia, attachment, and somatization (risk factors for AN [11, 43]) are related to mothers’ difficulty attuning with their daughters’ perception of academic performance, social complaints, aggressive behaviors, and externalizing problems. This underlines the involvement of mother–daughter dyssynchrony with other psychopathological risk factors of AN, supporting a multiple pathway interpretation of the role of dyssynchrony in this disorder.

The only significant difference between father–mother perception of daughter’s thought problems and the absence of relevant correlations with psychopathology suggests the greatest relevance of each parent’s relationship with the daughter for her suffering with respect to parents’ attunement on her perception [44].

This study represents the first attempt to measure parent–daughter emotional dyssynchrony and correlate it with the personality and psychopathology of adolescents affected with AN. The correlation pattern was wide, strong, and differentiated between the parents. Although it is not possible to evince any causal effect, the results showed that parental emotional dyssynchrony with AN adolescent daughters is highly connected with their personality traits and eating psychopathology. In fact, as recently underlined by Berking [45], psychopathology may be conceived as a maladaptive strategy to copy with undesired affective states. Since, as stated by Ratcliffe [46], the emotional regulation my utilize external “scaffolding” of emotions in particular when regulatory structure is loss, as it happens in adolescence. Improving parental dyssynchrony represents a relevant target for family interventions based on counseling [30] or family therapy [23, 28] to improve emotional regulation of adolescents affected with anorexia nervosa. In particular this could be even more relevant when parents display any kind of psychopathology, since it is well established that parent’s emotional dysregulation may severely affect mental health of the children [47]. The administration of the YSR and CBCL for the assessment of parent–son dyssynchrony can be useful for monitoring the effectiveness of family interventions. Future research should explore the prognostic value of parental dyssynchrony in the treatment of adolescents with AN and its application in tailoring therapeutic interventions.

Strengths and limits: the major strength is the exploration of a construct (emotional dyssynchrony) which has been theorized as relevant for attachment, parent–children interaction and children psychopathology development but poorly explored with validated instruments. Moreover, the findings are clear, statistically robust and consistent with previous literature and they evidence as the emotional dyssynchrony may represent a relevant therapeutic target.

The present is a cross-sectional study not providing information on the direction and causality of the correlations found. The measure adopted for dyssynchrony was extracted from two validated paired tests. However, it was applied for the first time in this study. The participant sample diagnostic distribution did not permit differentiation between restricter and binge-purging AN or between male and female AN. As it concerns the paternal subsample, the dropout rate was large, and thus less valid conclusions can be drawn. No control group was available; thus, the findings were not compared with the nonclinical adolescent population other than a literature one.

What is already known on this subject? It is known that emotional attunement is relevant for the attachment development and functioning in parent–children relationship. Emotional attunement is thought to influence the health and emotional regulation skills of the children.

What this study adds? It quantifies the emotional dyssynchrony in a sample of AN adolescents and their parents and supports a relationship between this construct and daughters’ psychopathology. The emotional dyssynchrony may be a specific target for family interventions to improve the care of anorexia nervosa or to prevent it.

## Data Availability

The data sets generated during and/or analysed during the current study are available from the corresponding author on reasonable request. The data that support the findings of this study are not openly available due to reasons of sensitivity and are available from the corresponding author upon reasonable request. Data are located in controlled access data storage at University of Torino.
